# Retraction: A wireless soil moisture sensor powered by solar energy

**DOI:** 10.1371/journal.pone.0195052

**Published:** 2018-03-23

**Authors:** Mingliang Jiang, Mouchao Lv, Zhong Deng, Guoliang Zhai, the PLOS ONE Editors

The authors and *PLOS ONE* Editors retract this article due to concerns about plagiarism and authorship issues.

This *PLOS ONE* article reports the development of a wireless soil moisture sensor, and validation data collected using soil samples under laboratory conditions [1]. However, the study rationale and sensor design reported in the *PLOS ONE* article by Jiang et al. overlaps substantially with a design reported by Yirui Sun et al. in *Computers and Electronics in Agriculture* [2]. There is substantial overlap in text, ideas, and experimental data and results presented in the *PLOS ONE* article and [2]. Furthermore, the following figures, table, and equations overlap with the related articles by Sun et al. [2, 3]:

*PLOS ONE* Fig 1 is adapted from Fig 1 of [2] and Fig 3 of [3].

*PLOS ONE* Fig 2 duplicates Fig 4 of [2].

*PLOS ONE* Fig 3 is adapted from Fig 4 of [3].

*PLOS ONE* Fig 4 is adapted from Fig 2 of [2].

*PLOS ONE* Table 3 duplicates Table 1 of [2].

*PLOS ONE* Equations 1–4 and related discussion overlap with Equations 4–7 of [3].

*PLOS ONE* Equation 5 overlaps with Equation 3 of [3]. The related articles by Sun et al. [2, 3] were not cited or discussed in the *PLOS ONE* article [1].

The authors agreed there is substantial overlap between the articles and that the *PLOS ONE* article does not provide attribution to earlier material used. In light of these concerns, the authors and *PLOS ONE* Editors retract this article.

ML requested the retraction, and all co-authors agreed. The authors apologize to the Academic Editor, reviewers, and readers.

GZ and ZD noted that they did not contribute to this research or preparation of the manuscript, and that they did not approve the submission to *PLOS ONE*. MJ claimed responsibility for the issues raised and indicated that he submitted the manuscript to *PLOS ONE* without the notification or consent of these two co-authors.

*PLOS ONE* notified the Chinese Academy of Agricultural Sciences about the plagiarism concerns and retraction.
